# Smoking and use of primary care services: findings from a population-based cohort study linked with administrative claims data

**DOI:** 10.1186/1472-6963-12-263

**Published:** 2012-08-18

**Authors:** Louisa R Jorm, Leah C Shepherd, Kris D Rogers, Fiona M Blyth

**Affiliations:** 1 Centre for Health Research, School of Medicine, University of Western Sydney, Locked Bag 1797, Penrith, NSW 2751, Australia; 2 The Sax Institute, Broadway, New South Wales, Australia; 3 Concord Clinical School, University of Sydney, Concord, New South Wales, Australia

## Abstract

**Background:**

Available evidence suggests that smokers have a lower propensity than others to use primary care services. But previous studies have incorporated only limited adjustment for confounding and mediating factors such as income, access to services and health status. We used data from a large prospective cohort study (the 45 and Up Study), linked to administrative claims data, to quantify the relationship between smoking status and use of primary care services, including specific preventive services, in a contemporary Australian population.

**Methods:**

Baseline questionnaire data from the 45 and Up Study were linked to administrative claims (Medicare) data for the 12-month period following study entry. The main outcome measures were Medicare benefit claimed for unreferred services, out-of-pocket costs (OOPC) paid, and claims for specific preventive services (immunisations, health assessments, chronic disease management services, PSA tests and Pap smears). Rate ratios with 95% confidence intervals were estimated using a hierarchical series of models, adjusted for predisposing, access- and health-related factors. Separate hurdle (two part) regression models were constructed for Medicare benefit and OOPC. Poisson models with robust error variance were used to model use of each specific preventive service.

**Results:**

Participants included 254,382 people aged 45 years and over of whom 7.3% were current smokers. After adjustment for predisposing, access- and health-related factors, current smokers were very slightly less likely to have claimed Medicare benefit than never smokers. Among those who claimed benefit, current smokers claimed similar total benefit, but recent quitters claimed significantly greater benefit, compared to never-smokers. Current smokers were around 10% less likely than never smokers to have paid any OOPC. Current smokers were 15-20% less likely than never smokers to use immunisations, Pap smears and prostate specific antigen tests.

**Conclusions:**

Current smokers were less likely than others to use primary care services that incurred out of pocket costs, and specific preventive services. This was independent of a wide range of predisposing, access- and health-related factors, suggesting that smokers have a lower propensity to seek health care. Smokers may be missing out on preventive services from which they would differentially benefit.

## Background

Smokers are at increased risk of many conditions that are amenable to prevention, early intervention and disease management in primary care, including hypertension, cardiovascular disease, diabetes, exacerbation of asthma and cervical cancer [1]. General practice-based brief interventions are among the most effective interventions for smoking cessation on a population basis [2]. And yet little is known about smokers’ use of primary care. The scant existing evidence comes from cohort studies in the United States (US) [3], Japan [4] and Korea [5] and a cross-sectional population survey in Canada [6]. All found that current smokers used fewer out-of-hospital services than never-smokers. The studies variously found that service use among previous smokers was slightly less than [4,5], similar to [6] or greater than [3] that of never smokers. The applicability of these findings to other contemporary populations and health systems is unknown. A small number of studies have investigated the use of specific preventive services according to smoking status. These have reported lower use by current smokers of services including Pap smears [7-9], mammograms [7-9], prostate specific antigen (PSA) tests [9], cholesterol screens [9], blood pressure checks, faecal occult blood tests [7,9], retinal examinations for diabetes [8] and influenza vaccinations [9]. Lower use of primary care services, in particular preventive services, by smokers is of concern because it may mean that opportunities for early intervention and secondary prevention are being missed.

Taken together, the available evidence suggests that smokers have a lower propensity than others to use primary care services. But previous studies have incorporated only limited adjustment for confounding and mediating factors such as income, access to services and health status. We used data from a large prospective cohort study, linked to administrative claims data, to quantify the relationship between smoking status and use of primary care services, including specific preventive services (immunisations, Pap smears, PSA tests, health assessments, chronic disease management), in a contemporary Australian population.

## Methods

The 45 and Up Study is a large-scale cohort involving 266,848 men and women aged 45 and over from New South Wales (NSW), Australia. Participants in the 45 and Up Study were randomly sampled from the database of Australia’s universal health insurance provider, Medicare Australia, which provides virtually complete coverage of the general population. Participants joined the Study by completing a baseline questionnaire (between February 2006 and April 2009) and giving signed consent for follow-up and linkage of their information to a range of health databases. The overall response rate was 18%. The Study is described in detail elsewhere [10]. Baseline questionnaire data from study participants were linked to Medicare claims data for the 12 months period following entry into the study using an encrypted version of the Medicare identification number. In order to identify and exclude participants who had died in this 12-month period, baseline questionnaire data were linked to individual death registration data from the NSW Registry of Births, Deaths and Marriages (to end of December 2009) using probabilistic methods.

All of the exposure variables used in this analysis were derived from self-reported data from the 45 and Up Study questionnaire (available at http://www.45andUp.org.au), apart from the measure of remoteness of residence, which was assigned according to the mean Accessibility Remoteness Index of Australia Plus (ARIA+) [11] score for the Postal Area of the participant’s residential address as recorded by Medicare. Variables were classified according to the groupings in Table 1, with an additional category for missing values.

**Table 1:** Characteristics of study participants according to smoking status

		**Current smoker N (%)**	**Previous smoker <5 yrs N (%)**	**Previous smoker** **5 + yrs N (%)**	**Never smoker** **N (%)**	**Total** **N (%)**
**Sex**	Male	9,061 (48.6)	5,273 (51.3)	47,003 (57.4)	55,991 (39.0)	117,328 (46.1)
	Female	9,595 (51.4)	5,011 (48.7)	34,867 (42.6)	87,581 (61.0)	137,054 (53.9)
**Age**	45 – 64 years	15,002 (80.4)	7,843 (76.3)	45,758 (55.9)	89,071 (62.0)	157,674 (62)
	65 – 79 years	3,182 (17.1)	2,052 (20.0)	27,038 (33.0)	40,091 (27.9)	72,363 (28.4)
	80 + years	472 (2.5)	389 (3.8)	9,074 (11.1)	14,410 (10.0)	24,345 (9.6)
**Country of birth**	Australia	13,874 (75.2)	7,476 (73.4)	59,526 (73.4)	110,045 (77.3)	190,921 (75.1)
	Overseas	4,564 (24.8)	2,710 (26.6)	21,552 (26.6)	32,275 (22.7)	61,101 (24)
LOTE*	No	16,691 (89.5)	9,290 (90.3)	75,556 (92.3)	128,865 (89.8)	230,402 (90.6)
	Yes	1,965 (10.5)	994 (9.7)	6,314 (7.7)	14,707 (10.2)	23,980 (9.4)
**Highest qualification**	University degree	2,485 (13.6)	1,660 (16.5)	17,318 (21.5)	37,315 (26.4)	58,778 (23.1)
	Post school qualification	6,042 (33.0)	3,525 (34.9)	28,533 (35.4)	42,954 (30.4)	81,054 (31.9)
	Higher school/leaving cert.	2,133 (11.6)	1,097 (10.9)	7,959 (9.9)	13,695 (9.7)	24,884 (9.8)
	School/intermediate cert.	4,374 (23.9)	2,306 (22.9)	16,804 (20.9)	32,688 (23.1)	56,172 (22.1)
	No qualification	3,299 (18.0)	1,501 (14.9)	9,926 (12.3)	14,726 (10.4)	29,452 (11.6)
**Annual household income**	$70,000 or more	3,051 (20.5)	2,219 (27.2)	18,822 (28.7)	36,423 (32.8)	60,515 (23.8)
	$40,000-$69,999	3,374 (22.6)	1,950 (23.9)	14,948 (22.8)	25,050 (22.5)	45,322 (17.8)
	$20,000-$39,999	3,382 (22.7)	1,754 (21.5)	15,474 (23.6)	24,048 (21.6)	44,658 (17.6)
	Less then $20,000	5,092 (34.2)	2,229 (27.3)	16,295 (24.9)	25,673 (23.1)	49,289 (19.4)
**Private health insurance**	None	5,270 (28.2)	2,467 (24.0)	13,600 (16.6)	21,835 (15.2)	43,172 (17)
	Health care card	5,682 (30.5)	2,453 (23.9)	15,277 (18.7)	21,351 (14.9)	44,763 (17.6)
	Private no extras	1,626 (8.7)	1,167 (11.3)	11,594 (14.2)	22,293 (15.5)	36,680 (14.4)
	Private with extras	5,892 (31.6)	4,067 (39.5)	39,277 (48.0)	76,264 (53.1)	125,500 (49.3)
	Veterans health care	186 (1.0)	130 (1.3)	2,122 (2.6)	1,829 (1.3)	4,267 (1.7)
**Working status**	Not working	8,144 (44.5)	4,534 (44.8)	43,319 (53.5)	68,850 (48.9)	124,847 (49.1)
	Part time	3,674 (20.1)	1,940 (19.2)	14,738 (18.2)	28,125 (20.0)	48,477 (19.1)
	Full time	6,470 (35.4)	3,656 (36.1)	22,902 (28.3)	43,866 (31.1)	76,894 (30.2)
**Remoteness of residence**	Major Cities	7,581 (40.6)	4,249 (41.3)	35,615 (43.5)	66,555 (46.4)	114,000 (44.8)
	Inner Regional	6,744 (36.2)	3,820 (37.2)	30,103 (36.8)	49,044 (34.2)	89,711 (35.3)
	Outer Regional	3,780 (20.3)	1,943 (18.9)	14,533 (17.8)	25,184 (17.6)	45,440 (17.9)
	Remote	545 (2.9)	266 (2.6)	1,566 (1.9)	2,690 (1.9)	5,067 (2)
**Alcoholic drinks/week**	0 - 1 drinks	6,889 (37.8)	3,550 (35.0)	21,878 (27.0)	62,025 (44.2)	94,342 (37.1)
	2 - 4 drinks	2,128 (11.7)	1,241 (12.3)	10,910 (13.5)	23,844 (17.0)	38,123 (15)
	5 - 10 drinks	3,659 (20.1)	2,186 (21.6)	21,935 (27.1)	34,175 (24.4)	61,955 (24.4)
	11 + drinks	5,571 (30.5)	3,153 (31.1)	26,218 (32.4)	20,234 (14.4)	55,176 (21.7)
**Psychological distress**	Low	11,740 (64.4)	6,971 (69.3)	62,942 (78.7)	112,094 (80.2)	193,747 (76.2)
	Moderate	3,506 (19.2)	1,906 (18.9)	11,833 (14.8)	19,491 (13.9)	36,736 (14.4)
	High	1,882 (10.3)	813 (8.1)	3,845 (4.8)	5,969 (4.3)	12,509 (4.9)
	Very High	1,104 (6.1)	369 (3.7)	1,314 (1.6)	2,198 (1.6)	4,985 (2)
**Functional limitation**	No limitation	4,899 (29.5)	2,662 (28.6)	22,203 (29.9)	46,648 (36.1)	76,412 (30)
	Minor limitation	2,588 (15.6)	1,476 (15.8)	12,263 (16.5)	21,968 (17.0)	38,295 (15.1)
	Mild limitation	3,020 (18.2)	1,665 (17.9)	14,685 (19.8)	23,293 (18.0)	42,663 (16.8)
	Moderate limitation	2,901 (17.5)	1,663 (17.8)	13,451 (18.1)	20,169 (15.6)	38,184 (15)
	Severe limitation	3,208 (19.3)	1,851 (19.9)	11,666 (15.7)	17,223 (13.3)	33,948 (13.3)
**Self rated health**	Excellent	1,382 (7.7)	957 (9.7)	11,099 (14.0)	23,978 (17.3)	37,416 (14.7)
	Very good	5,015 (28.0)	3,054 (30.9)	29,019 (36.6)	54,463 (39.3)	91,551 (36)
	Good	7,158 (40.0)	3,793 (38.3)	27,553 (34.8)	44,430 (32.0)	82,934 (32.6)
	Fair	3,537 (19.7)	1,702 (17.2)	9,782 (12.4)	13,731 (9.9)	28,752 (11.3)
	Poor	818 (4.6)	389 (3.9)	1,744 (2.2)	2,057 (1.5)	5,008 (2)
**Comorbidities count**	0	11,225 (61.2)	5,571 (54.7)	41,382 (51.1)	82,487 (57.9)	140,665 (55.3)
	1	4,934 (26.9)	3,094 (30.4)	25,461 (31.4)	41,437 (29.1)	74,926 (29.5)
	2	1,538 (8.4)	1,094 (10.7)	9,996 (12.3)	13,356 (9.4)	25,984 (10.2)
	3 or more	655 (3.6)	432 (4.2)	4,218 (5.2)	5,117 (3.6)	10,422 (4.1)
	**Total**	**18,658 (7.3)**	**10,284 (4.0)**	**81,870 (32.2)**	**143,572 (56.4)**	**254,382 (100)**

*Language other than English spoken at home.

Regarding smoking status, participants were asked: ‘Have you ever been a regular smoker?’, (if yes) ‘How old were you when you started smoking regularly?’, ‘Are you a regular smoker now?’ and (if no) ‘How old were you when you stopped smoking regularly?’. Participants were classified into four groups: never smokers, previous smokers who quit within the past 5 years (‘recent quitters’), previous smokers who quit 5 or more years ago and current smokers.

Functional limitation was measured using the Medical Outcomes Study Physical Functioning Scale [12] (equivalent to the physical functioning sub-score from the SF-36), grouped into five categories: no (score 100), minor (95 – 99), mild (85 – 94), moderate (60 – 84) and severe (0 – 59) limitation. Psychological distress was measured using the Kessler 10 score [13], grouped into four categories: low (score 0 – 15), moderate (16 – 21), high (22 – 29) and very high (30 or higher) psychological distress. Co-morbidities (cancers, diabetes, arthritis, heart disease, stroke, blood clot, asthma, enlarged prostate and Parkinsons disease) were identified according to the response to the question “Has a doctor ever told you you have any of the following…”.

Medicare Australia processes claims for subsidised medical and diagnostic services provided to Australian citizens by registered medical and other practitioners through the Medicare Benefits Scheme (MBS). Specific MBS services are coded using a system of Item Numbers listed in the Medical Benefits Schedule. Our analysis used two cost variables from MBS claims data: provider charge (the amount the provider charged for the service) and benefit paid (the benefit paid by Medicare to the claimant), and we defined out-of-pocket costs (OOPC) as the difference between these two amounts. All costs were adjusted to 2007/08 dollars using the total health price index published by the Australian Institute of Health and Welfare [14].

We explored five different measures of primary care use, all for the 12-month period after the date of entry into the 45 and Up Study: (i) whether participants had claimed Medicare benefit for unreferred services (services that were not been referred to that practitioner by another medical practitioner, which equate to primary care services provided by general practitioners [GPs] and practice nurses); (ii) for those participants claiming Medicare benefit, total amount claimed; (iii) for those participants claiming Medicare benefit, whether they had incurred OOPC for unreferred services; (iv) for those participants incurring OOPC, total costs incurred; (v) whether participants had made claims for the following specific preventive services (where these were not provided by a medical specialist): immunisations, health assessments, chronic disease management services, PSA tests (males) and Pap smears (females).

Rate ratios (RR) with 95% confidence intervals (CI) for each of these measures were estimated using a hierarchical series of models, guided by the Anderson and Newman theoretical framework for health services utilisation [15]. Model 1 was adjusted for age only. Model 2 was adjusted for age plus the following predisposing and access-related factors: highest level of education, country of birth, language other than English spoken at home, annual household income, remoteness of residence, working status, private health insurance, alcoholic drinks per week. Model 3 included the same variables as Model 2, with the addition of the following health-related factors: self-rated health, functional capacity, psychological distress, comorbidities count. All models were stratified by sex. Women who indicated that they had had a hysterectomy were excluded from the Pap smear models only.

Separate hurdle (or two part) regression models were constructed for Medicare benefit and OOPC. These models separated use or non-use of services from volume of use (total cost) among service users and assumed that these were generated by two systematically different statistical processes: a binomial distribution for service use or non-use, and a gamma distribution for all non-zero cost [16,17] Poisson models with robust error variance [18] were used to model use of each specific preventive service. All analysis was carried out in SAS V9 [19].

This study was approved by the Department of Health and Ageing Ethics Committee (1/2005). The 45 and Up Study has primary ethical approval from the University of New South Wales Human Research Ethics Committee (HREC 05035).

## Results

After excluding 45 and Up Study participants who died within 12 months of entering the study (n = 2,067), whose entry date into the study was after 31 December 2008 and who therefore did not have 12 months of follow-up (n = 3,656), who had an invalid age (n = 6), who lived outside of NSW (n = 72), were not linked with Medicare data (n =1,190), or had insufficient self-report information to determine smoking status (n = 2,475), records for 254,382 participants were available for analysis. These participants claimed for 2,076,310 unreferred attendances in the 12 months following entry into the 45 and Up Study, of which 297,175 (6.9%) were for preventive services.

The characteristics of participants are shown in Table 1. Compared with never smokers, current smokers were more likely to be female, younger, separated, divorced or single, less educated, have a lower annual income, and hold a health care card. They were more likely to work full time, to live in outer regional or remote areas and to hold no private health insurance. Current smokers generally reported poorer mental health, self-rated health and physical functioning than previous and never smokers, but they had fewer comorbidities than previous smokers. Both current and previous smokers consumed more standard drinks per week than never smokers. Previous smokers had similar characteristics to never smokers overall.

Table 2 presents the results of models that explored Medicare benefit claims and OOPC paid. In the age-adjusted-only analysis (Model 1), after adjusting for access-related and predisposing factors (Model 2) and after adjusting also for health-related factors (Model 3), both male and female current smokers were slightly less likely to have claimed Medicare benefit for unreferred attendances than never smokers, while previous smokers were slightly more likely to have claimed benefit. The relevant effect sizes, however, were small.

**Table 2:** Medicare benefit claims and out-of-pocket costs in 12 months following study entry, according to smoking status

**Medicare benefit**	**% Participants**	**% Claimed benefit**	**RR (95% CI)**						**Median benefit claimed ($)**
			Model 1*		**Model 2**†		**Model 3**‡		
			**Claimed benefit**	**Cost claimed**§	**Claimed benefit**	**Cost claimed**§	**Claimed benefit**	**Cost claimed**§	
**Males (N = 117,328)**									
Never smoker	47.7	91.9	Reference	Reference	Reference	Reference	Reference	Reference	207.55
Previous smoker < 5 yrs	4.5	92.3	1.01 (1.00 - 1.02)	1.29 (1.25 - 1.33)	1.01 (1.00 - 1.02)	1.19 (1.16 - 1.22)	1.00 (0.99 - 1.01)	1.09 (1.06 - 1.12)	245.24
Previous smoker 5+ yrs	40.1	93.0	1.01 (1.00 - 1.01)	1.13 (1.12 - 1.14)	1.01 (1.01 - 1.01)	1.12 (1.10 - 1.13)	1.01 (1.00 - 1.01)	1.06 (1.05 - 1.07)	268.40
Current smoker	7.7	89.1	0.98 (0.97 - 0.98)	1.25 (1.22 - 1.28)	0.97 (0.97 - 0.98)	1.08 (1.06 - 1.11)	0.97 (0.96 - 0.97)	1.01 (0.99 - 1.03)	223.95
**Total**	100	92.1							233.92
**Females (N = 137,054)**									
Never smoker	63.9	95.2	Reference	Reference	Reference	Reference	Reference	Reference	255.75
Previous smoker < 5 yrs	3.6	95.6	1.01 (1.00 - 1.01)	1.23 (1.20 - 1.27)	1.01 (1.00 - 1.01)	1.19 (1.16 - 1.22)	1.00 (1.00 - 1.01)	1.09 (1.06 - 1.11)	277.05
Previous smoker 5 + yrs	25.4	95.4	1.00 (1.00 - 1.01)	1.05 (1.04 - 1.07)	1.01 (1.00 - 1.01)	1.10 (1.09 - 1.11)	1.00 (1.00 - 1.01)	1.06 (1.05 - 1.07)	259.85
Current smoker	7.7	93.9	0.99 (0.98 – 0.99)	1.17 (1.15 - 1.20)	0.99 (0.98 - 0.99)	1.10 (1.08 - 1.12)	0.98 (0.98 - 0.99)	1.00 (0.98 - 1.02)	246.48
**Total**	100	95.2							258.2
**Out of pocket costs (OOPC)**	**% Participants**§	**% Paid OOPC**§	**RR (95% CI)**						**Median OOPC paid**§ **($)**
			Model 1*		**Model 2**†		**Model 3**‡		
			**Paid OOPC**§	**Amount paid**||	**Paid OOPC**§	**Amount paid**||	**Paid OOPC**§	**Amount paid**||	
**Males (N = 117,328)**									
Never smoker	47.7	50.1	Reference	Reference	Reference	Reference	Reference	Reference	50.80
Previous smoker < 5 yrs	4.5	44.0	0.83 (0.80 - 0.85)	1.02 (0.98 - 1.06)	0.93 (0.91 - 0.96)	1.07 (1.03 - 1.12)	0.95 (0.92 - 0.98)	1.03 (1.00 - 1.07)	52.05
Previous smoker 5+ yrs	40.1	45.3	0.96 (0.95 - 0.98)	1.04 (1.02 - 1.06)	0.99 (0.97 – 1.00)	1.06 (1.04 - 1.08)	0.99 (0.98 - 1.01)	1.04 (1.02 - 1.05)	53.45
Current smoker	7.7	37.3	0.69 (0.67 - 0.71)	0.91 (0.88 - 0.94)	0.86 (0.84 - 0.88)	0.98 (0.95 - 1.02)	0.87 (0.85 - 0.90)	0.94 (0.91 - 0.98)	44.88
**Total**	100	46.9							51.55
**Females (N = 137,054)**									
Never smoker	63.9	50.6	Reference	Reference	Reference	Reference	Reference	Reference	56.55
Previous smoker < 5 yrs	3.6	49.7	0.90 (0.87 - 0.92)	1.00 (0.96 - 1.03)	0.96 (0.94 - 0.99)	1.05 (1.01 - 1.09)	0.98 (0.95 - 1.01)	1.01 (0.98 - 1.05)	56.90
Previous smoker 5 + yrs	25.4	54.4	1.05 (1.03 - 1.06)	1.05 (1.03 - 1.07)	1.02 (1.00 - 1.03)	1.06 (1.04 - 1.08)	1.02 (1.01 - 1.03)	1.04 (1.03 - 1.06)	58.85
Current smoker	7.7	42.82	0.76 (0.74 - 0.78)	0.93 (0.90 - 0.96)	0.87 (0.85 - 0.89)	1.01 (0.98 - 1.04)	0.88 (0.86 - 0.90)	0.97 (0.94 – 1.00)	51.60
**Total**	100	51.1							56.90

*Model 1: adjusted for age.

†Model 2: adjusted for age, highest level of education, country of birth, language other than English spoken at home, annual household income, remoteness of residence, working status, private health insurance, alcoholic drinks per week.

‡Model 3: adjusted as for Model 2, plus self-rated health, functional capacity, psychological distress, comorbidities count.

§Includes only participants who claimed Medicare benefit.

||Includes only participants who paid OOPC.

In the age-adjusted-only analysis (Model 1), among those participants who had claimed Medicare benefit, male and female current smokers claimed greater total benefit than never smokers, but this effect was attenuated after adjustment for access-related and predisposing factors (Model 2) and disappeared after adjusting also for health-related factors (Model 3). Ex-smokers also claimed significantly greater benefit than never smokers, but this effect persisted after all adjustments and was stronger among men and women who had quit in the last 5 years (‘recent quitters’) compared to those who had quit prior to this.

Among participants who had claimed Medicare benefit, current smokers of both sexes were less likely than never smokers in the age-adjusted-only analysis (Model 1) to have paid any OOPC in the 12 months following study entry. This effect was attenuated after further adjustment, but remained significant in the fully adjusted model (Model 3). Recent quitters were marginally less likely to have paid OOPC than never smokers, but this small effect attenuated even further with adjustment. Previous smokers who had quit 5 or more years ago, on the other hand, were slightly more likely to have paid OOPC than never smokers, but again this effect was small and attenuated with adjustment.

Among those participants who had paid OOPC in the past 12 months, current smokers paid 20-25% less total OOPC than never smokers in the age-adjusted-only analysis (Model 1), but this effect disappeared with adjustment for access, predisposing and health-related factors (Models 2 and 3). Previous smokers paid similar total OOPC to never smokers.

Table 3 presents the result of analysis of use of preventive services. After adjusting for age, current smokers were less likely than never smokers to use immunisations, health assessments (males only), pap smears and PSA tests (Model 1). Adjusting for access and predisposing factors (Model 2) and health-related factors (Model 3) had little effect on these associations. In the age-adjusted-only analysis, (Model 1) current smokers were more likely than never smokers to use chronic disease management items. However, these effects attenuated with adjustment for access and predisposing factors (Model 2) and disappeared with adjustment for health-related factors (Model 3).

**Table 3:** Use of specific preventive services in 12 months following study entry, according to smoking status

**Males**	**PSA test**				**Immunization**			
	**N (%)**	**RR (95% CI)**			**N (%)**	**RR (95% CI)**		
		Model 1*	**Model 2**†	**Model 3**‡		Model 1*	**Model 2**†	**Model 3**‡
**Never smoker**	21149 (37.7)	Reference	Reference	Reference	12719 (22.7)	Reference	Reference	Reference
**Previous smoker < 5 yrs**	1968 (37.3)	0.97 (0.94 - 1.01)	0.97 (0.94 - 1.01)	0.99 (0.95 - 1.03)	1127 (21.3)	1.08 (1.03 - 1.14)	1.04 (0.99 - 1.1)	1.01 (0.96 - 1.06)
**Previous smoker 5 + yrs**	17665 (37.6)	1.01 (0.99 - 1.02)	1 (0.98 - 1.01)	1.01 (0.99 - 1.02)	13386 (28.5)	1.09 (1.07 - 1.11)	1.06 (1.04 - 1.08)	1.03 (1.01 - 1.05)
**Current smoker**	2883 (31.8)	0.84 (0.81 - 0.86)	0.84 (0.82 - 0.87)	0.85 (0.83 - 0.88)	1406 (15.5)	0.83 (0.79 - 0.87)	0.79 (0.75 - 0.83)	0.78 (0.75 - 0.82)
**Total**	43,665 (37.2)				28,638 (24.4)			
	**Health assessment**				**Chronic disease management**			
	**N (%)**	**RR (95% CI)**			**N (%)**	**RR (95% CI)**		
		Model 1*	**Model 2**†	**Model 3**‡		Model 1*	**Model 2**†	**Model 3**‡
**Never smoker**	5311 (9.5)	Reference	Reference	Reference	10783 (19.3)	Reference	Reference	Reference
**Previous smoker < 5 yrs**	341 (6.5)	0.96 (0.86 - 1.06)	0.93 (0.84 - 1.03)	0.91 (0.82 - 1.01)	1374 (26.1)	1.5 (1.43 - 1.58)	1.3 (1.24 - 1.37)	1.15 (1.09 - 1.2)
**Previous smoker 5 + yrs**	5386 (11.5)	0.96 (0.93 - 0.99)	0.97 (0.94 - 1.01)	0.96 (0.93 - 0.99)	12367 (26.3)	1.23 (1.2 - 1.26)	1.19 (1.16 - 1.22)	1.09 (1.06 - 1.11)
**Current smoker**	483 (5.3)	0.88 (0.8 - 0.96)	0.84 (0.77 - 0.92)	0.83 (0.76 - 0.91)	2003 (22.1)	1.33 (1.28 - 1.39)	1.06 (1.02 - 1.11)	1 .00 (0.96 - 1.04)
**Total**	11,521 (9.8)				26,527 (22.6)			
**Females**	**Pap smear**§				**Immunization**			
	**N (%)**	**RR (95% CI)**			**N (%)**	**RR (95% CI)**		
		Model 1*	**Model 2**†	**Model 3**‡		Model 1*	**Model 2**†	**Model 3**‡
**Never smoker**	29528 (47.2)	Reference	Reference	Reference	22641 (25.9)	Reference	Reference	Reference
**Previous smoker < 5 yrs**	1677 (47.7)	0.89 (0.86 - 0.92)	0.91 (0.88 - 0.94)	0.93 (0.9 - 0.97)	1131 (22.6)	1.05 (1–1.1)	1.02 (0.97 - 1.07)	0.99 (0.94 - 1.04)
**Previous smoker 5 + yrs**	12300 (49.0)	1 (0.98 - 1.01)	0.98 (0.97 - 0.99)	0.99 (0.97 – 1.00)	9030 (25.9)	1.04 (1.02 - 1.07)	1.05 (1.03 - 1.07)	1.03 (1.01 - 1.05)
**Current smoker**	2907 (42.9)	0.78 (0.76 - 0.81)	0.82 (0.80 - 0.84)	0.84 (0.82 - 0.86)	1655 (17.2)	0.85 (0.81 - 0.89)	0.82 (0.78 - 0.85)	0.81 (0.77 - 0.84)
**Total**	46,412 (47.4)				34,457 (25.1)			
	**Health assessment**				**Chronic disease management**			
	**N (%)**	**RR (95% CI)**			**N (%)**	**RR (95% CI)**		
		Model 1*	**Model 2**†	**Model 3**‡		Model 1*	**Model 2**†	**Model 3**‡
**Never smoker**	8924 (10.2)	Reference	Reference	Reference	19591 (22.4)	Reference	Reference	Reference
**Previous smoker < 5 yrs**	324 (6.5)	1.04 (0.94 - 1.15)	1.02 (0.92 - 1.13)	0.99 (0.89 - 1.1)	1326 (26.5)	1.41 (1.35 - 1.48)	1.31 (1.25 - 1.37)	1.15 (1.1 - 1.21)
**Previous smoker 5 + yrs**	2951 (8.5)	0.97 (0.94 - 1.01)	0.99 (0.95 - 1.02)	0.97 (0.94 - 1.01)	7930 (22.8)	1.07 (1.04 - 1.09)	1.14 (1.11 - 1.16)	1.07 (1.04 - 1.09)
**Current smoker**	581 (6.1)	1.13 (1.04 - 1.22)	1.09 (1.01 - 1.18)	1.06 (0.98 - 1.14)	2110 (22.0)	1.23 (1.19 - 1.28)	1.08 (1.04 - 1.13)	0.98 (0.94 - 1.02)
**Total**	12,780 (9.3)				30,957 (22.5)			

*Model 1: adjusted for age.

†Model 2: adjusted for age, highest level of education, country of birth, language other than English spoken at home, annual household income, remoteness of residence, working status, private health insurance, alcoholic drinks per week.

‡Model 3: adjusted as for Model 2, plus self-rated health, functional capacity, psychological distress, comorbidities count.

§Excludes women who reported having had a hysterectomy (n = 38,574).

Previous smokers who had quit more than 5 years ago were similar to never smokers in terms of their use of immunisations, health assessments, Pap smears and PSA tests, while recent quitters were more similar to current smokers. However, previous smokers, especially recent quitters, were more likely than never smokers to use chronic disease management items, effects which remained significant after all adjustments.

## Discussion

We found that current smokers were less likely than others to use primary care services overall, primary care services that they had to pay for themselves, and specific preventive services, and that these effects persisted after adjusting for predisposing and access-related factors including income, level of education and region of residence, and for measures of physical and mental health status. Although the effect sizes we demonstrated were small, they translate into considerable numbers of primary care services foregone by smokers at the population level, even in a country such as Australia in which tobacco control efforts have been highly successful.

In terms of overall use of primary care services, our findings were consistent with those of three previous cohort studies in diverse settings that used a variety of measures of service use [3-5]. Of these, the US study [3] is now more than 20 years old and was conducted within a health maintenance organisation, while the Japanese [4] and Korean [5] studies were in populations with very high male smoking rates (55% and 58% respectively) and low female smoking rates (9%,<1%). While the authors of previous studies have speculated that lower levels of care-seeking behaviour in smokers might underlie their patterns of use, our study was able to explore this issue further by incorporating comprehensive adjustment for potential confounding and mediating factors. We found that smokers’ lower levels of service use persisted despite these adjustments, suggesting that they indeed had a lower propensity to seek care. Smokers’ lower use of services that incurred OOPC, regardless of their socioeconomic status, was also consistent with lower levels of health-seeking behaviours.

We found that previous smokers, especially more recent quitters, were more likely than never smokers to use primary care services, and claimed significantly greater benefit. This was in contrast to findings from Japan [4] and Korea [5], where previous smokers used slightly less out-of-hospital services than never-smokers, but was consistent with US findings [3]. In terms of use of specific preventive services, we found that recent quitters were generally similar to current smokers, while smokers who had quit more than 5 years ago were similar to never smokers. However, previous smokers, especially recent quitters, were more likely than either current or never smokers to use Medicare chronic disease management items. These items enable GPs to plan and coordinate the health care of patients with chronic medical conditions, including asthma, cancer, cardiovascular disease, diabetes mellitus and stroke. Their high use by previous smokers is indicative of high rates of chronic disease. This suggests that a ‘healthy smoker’ effect (whereby sick smokers selectively quit smoking at greater rates than healthy smokers) was operating. However, this effect did not explain the lower use of primary care services among current smokers compared with never smokers in our study. Compared with never smokers, current smokers had higher levels of functional limitation, psychological distress and poor self-rated health, and their lower use of services persisted after adjusting for multiple measures of physical and mental health status.

Our findings regarding lower rates of use by smokers of specific preventive services (Pap smears, PSA tests, immunisations, health checks) were consistent with those of the small number of previous studies that have explored this issue [3,7-9]. The magnitude of these effects was comparable across studies, and across various types of preventive services, with use by current smokers around 20% lower than for never smokers. Although these effect sizes might seem relatively small, they translate into large numbers at the population level. Based on age-specific smoking prevalence in 2007 [20], our results imply that approximately 70,000 Pap smears and 150,000 immunisations are forgone annually by Australian smokers aged 45 years and over. This is of particular concern because female smokers are at approximately two-fold increased risk of cervical cancer [1], and smokers of both sexes are at increased risk of complications of influenza infection and of invasive pneumococcal disease [1]. Although the risks of PSA testing in healthy men may outweigh its benefits [21], and the cost-effectiveness of health checks provided through Medicare is unknown, lower use of both of these services by male smokers again points to lower levels of health seeking behaviours.

So why don’t smokers ‘look after themselves’? Numerous studies have found evidence of an optimistic bias in relation to smoking, such that smokers tend to see the risks of smoking as lower for themselves than for other smokers [22]. Fewer smokers than ex-smokers accept that smoking causes disease, and smokers also maintain beliefs that exempt them from personalising widespread acceptance that smoking harms health [23]. Such attitudes might translate into denial of other health risks (such as cervical cancer or vaccine-preventable illness), denial of illness itself, and to delays in seeking care. Additionally, there is evidence that smokers are deterred from seeking health care through embarrassment and fear of discrimination [24] fear of being blamed by health professionals for their own ill-health [25,26], and feelings of guilt about not having quit smoking [27].

Our study had some limitations. The 45 and Up Study had a response rate of 18% (similar to other cohort studies of this nature) and the prevalence of regular smoking (weighted for age, sex and remoteness) (7.5%) was lower than the figure for daily smoking from the most comparable population survey (12%) [28]. However, the large sample size of the 45 and Up Study provides substantial heterogeneity and in these circumstances RRs calculated from internal comparisons within a cohort remain valid [29]. Moreover, empirical data from the 45 and Up Study show that RRs relating to smoking in the cohort are very similar to those calculated using ‘representative’ population survey data [28]. Medicare claims data contain only limited information about the reasons for service use and only some preventive services have specific MBS item numbers. As a result we could not examine services such as mammographic and colorectal screening, blood pressure and blood cholesterol checks and— importantly—smoking cessation interventions. We could not identify specific types of immunisation.

However, as the largest prospective study of its subject to date, and the only one to incorporate comprehensive adjustment for a wide range of predisposing-, access- and health-related factors, our study has provided new evidence that is highly pertinent to tobacco control efforts. It suggests that smokers have a lower propensity than others to seek health care, to use services that they have to pay for, and to use preventive services. They may be missing out on preventive services, such as Pap smears and immunisations, from which they would differentially benefit. Encouragingly, though, we found that around 90% of smokers used primary care at least once in a 12-month period. The challenge for health funders, policymakers and providers is to capitalise on these opportunities for prevention, while not inadvertently further deterring smokers from seeking care.

## Conclusion

Current smokers were less likely than others to use primary care services that incurred out of pocket costs, and specific preventive services. This was independent of a wide range of predisposing, access- and health-related factors, suggesting that smokers have a lower propensity to seek health care. Smokers may be missing out on preventive services from which they would differentially benefit.

## Competing interests

There are no competing interests to declare.

## Authors’ contributions

LJ participated in the design of the study, oversaw data analysis and drafted the manuscript. LS and KR participated in the design of the study, performed the statistical analysis and helped to draft the manuscript. FB participated in the design of the study, oversaw data analysis and helped to draft the manuscript. All authors read and approved the final manuscript.

## Pre-publication history

The pre-publication history for this paper can be accessed here:

http://www.biomedcentral.com/1472-6963/12/263/prepub

## References

[B1] U.S. Department of Health and Human Services The Health Consequences of Smoking: A Report of the Surgeon General 2004 U.S. Department of Health and Human Services, Centers for Disease Control and Prevention, National Center for Chronic Disease Prevention and Health Promotion, Office on Smoking and Health, Atlanta

[B2] Stead LF Bergson G Lancaster T Physician advice for smoking cessation Cochrane Database Syst Rev 2008 Art. No.: CD000165 Issue 2 doi: 10.1002/14651858.CD000165.pub3 10.1002/14651858.CD000165.pub3 18425860

[B3] Vogt T Schweitzer S Medical costs of cigarette smoking in a health maintenance organization Am J Epidemiol 1985 122 1060 1066 4061440 10.1093/oxfordjournals.aje.a114187

[B4] Izumi Y Tsuji I Ohkubo T Kuwahara A Nishino Y Hisamichi S Impact of smoking habit on medical care use and its costs: a prospective observation of National Health Insurance beneficiaries in Japan Int J Epidemiol 2001 30 616 621 10.1093/ije/30.3.616 11416093

[B5] Kang HY Kim HJ Park TK Jee SH Nam CM Park HW Economic burden of smoking in Korea Tob Control 2003 12 37 44 10.1136/tc.12.1.37 12612360 PMC1759072

[B6] Asada Y Kephart G Equity in health services use and intensity of use in Canada BMC Health Serv Res 2007 7 41 10.1186/1472-6963-7-41 17349059 PMC1829158

[B7] Chao A Paganini-Hill A Ross RK Henderson BE Use of preventive care by the elderly Prev Med 1987 16 710 722 10.1016/0091-7435(87)90053-3 3684980

[B8] Ramsey SD Cheadle AD Neighbor WE Gore E Temple P Staiger T Goldberg HI Relative impact of patient and clinic factors on adherence to primary care preventive service guidelines: an exploratory study Med Care 2001 39 979 989 10.1097/00005650-200109000-00008 11502955

[B9] Green CA Polen MR Leo MC Perrin NA Anderson BM Weisner CM Drinking patterns, gender and health II: Predictors of preventive service use Addict Res Theory 2010 18 143 159 10.3109/16066350903398494 PMC3694481 23814545

[B10] Banks E Redman S Jorm L Armstrong B Bauman A Beard J Beral V Byles J Corbett S Cumming R Harris M Sitas F Smith W Taylor L Wutzke S Lujic S Cohort Profile: the 45 and Up Study Int J Epidemiol 2008 37 941 947 17881411 10.1093/ije/dym184 PMC2557061

[B11] Australian Institute of Health and Welfare Rural Regional and Remote Health: A guide to remoteness classifications 2004 Australian Institute of Health and Welfare, Canberra

[B12] Stewart AL Kamberg CJ Stewart AL, Ware J Physical functioning measures Measuring functioning and well-being: the Medical Outcomes Study approach 1992 Duke University Press, Durham, NC

[B13] Kessler R Mroczek D Final version of our never specific psychological distress scale [memo dated 3/10/94] 1994 Survey Research Center of the Institute for Social Research: University of Michigan, Ann Arbor, Mich

[B14] Australian Institute of Health and Welfare Health expenditure Australia 2007–08. Health and welfare expenditure series no 37 Cat no HWE 46 2009 AIHW, Canberra

[B15] Andersen R Newman JF Societal and individual determinants of medical care utilization in the United States Milbank Mem Fund Q Health Soc 1973 51 95 124 10.2307/3349613 4198894

[B16] Buntin MB Zaslavsky AM Too much ado about two-part models and transformation? Comparing methods of modeling Medicare expenditures J Health Econ 2004 23 3 525 542 10.1016/j.jhealeco.2003.10.005 15120469

[B17] Kenny P Hall J King M Lancsar E Sources of variation in the costs of health care for asthma patients in Australia J Health Serv Res Policy 2009 14 3 33 140 10.1258/jhsrp.2008.008078 19541871

[B18] Zou G A modified poisson regression approach to prospective studies with binary data Am J Epidemiol 2004 159 702 706 10.1093/aje/kwh090 15033648

[B19] SAS Institute Inc SAS Version 9.2 2007 NC, Cary

[B20] Population Health Division The health of the people of New South Wales - Report of the Chief Health Officer 2011 NSW Department of Health, Sydney Available at: www.health.nsw.gov.au/publichealth/chorep/. Accessed 17 November

[B21] Chou R Croswell JM Dana T Bougatsos C Blazina I Fu R Gleitsmann K Koenig HC Lam C Maltz A Rugge JB Lin K Screening for prostate cancer: a review of the evidence for the U.S. Preventive Services Task Force Ann Intern Med 2011 155 762 771 21984740 10.7326/0003-4819-155-11-201112060-00375

[B22] Arnett JJ Optimistic bias in adolescent and adult smokers and nonsmokers Addict Behav 2000 25 625 632 10.1016/S0306-4603(99)00072-6 10972456

[B23] Chapman S Wong WL Smith W Self-exempting beliefs about smoking and health: differences between smokers and ex-smokers Am J Public Health 1993 83 215 219 10.2105/AJPH.83.2.215 8427326 PMC1694573

[B24] Burrows J Carlisle J They don’t want it ramming down their throats. Learning from the perspectives of current and ex-smokers with smoking-related illness to improve communication in primary care: a qualitative study Prim Health Care Res Dev 2010 11 206 214 10.1017/S1463423609990351

[B25] Richards H Reid M Watt G Victim-blaming revisited: a qualitative study of beliefs about illness causation, and responses to chest pain Fam Pract 2003 20 711 716 10.1093/fampra/cmg615 14701897

[B26] Green CA Polen MR Leo MC Janoff SL Anderson BM Weisner CM Perrin NA Drinking patterns, gender and health III: Avoiding versus seeking health care Addict Research Theory 2010 18 160 180 10.3109/16066350903398502 PMC3686530 23795149

[B27] Butler CC Pill R Stott NCH Qualitative study of patients’ perceptions of doctors’ advice to quit smoking: implications for opportunistic health promotion Br Med J 1998 316 1878 1881 10.1136/bmj.316.7148.1878 9632409 PMC28587

[B28] Mealing NM Banks E Jorm LR Steel DG Clements MS Rogers KD Investigation of relative risk estimates from studies of the same population with contrasting response rates and designs BMC Med Res Methodol 2010 10 26 10.1186/1471-2288-10-26 20356408 PMC2868856

[B29] Ponsonby A Dwyer T Couper D Is this finding relevant? Generalisation and epidemiology Aust N Z J Public Health 1996 20 54 56 10.1111/j.1467-842X.1996.tb01336.x 8799067

